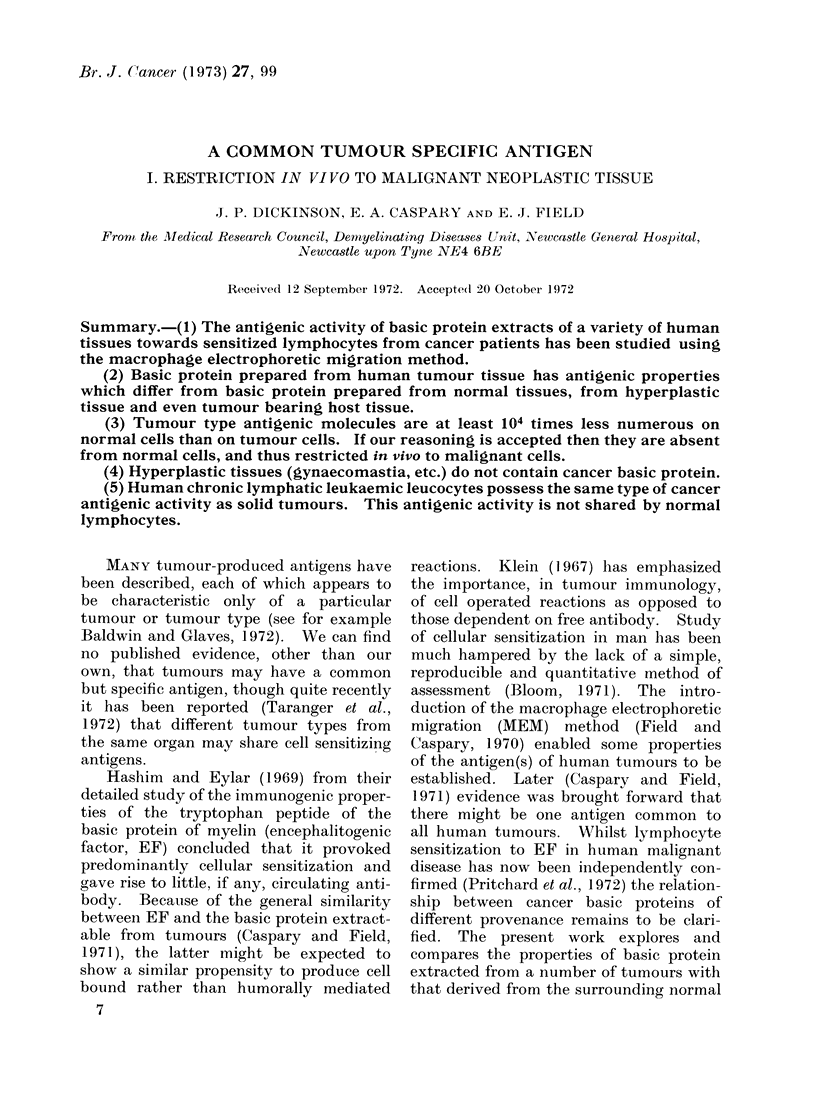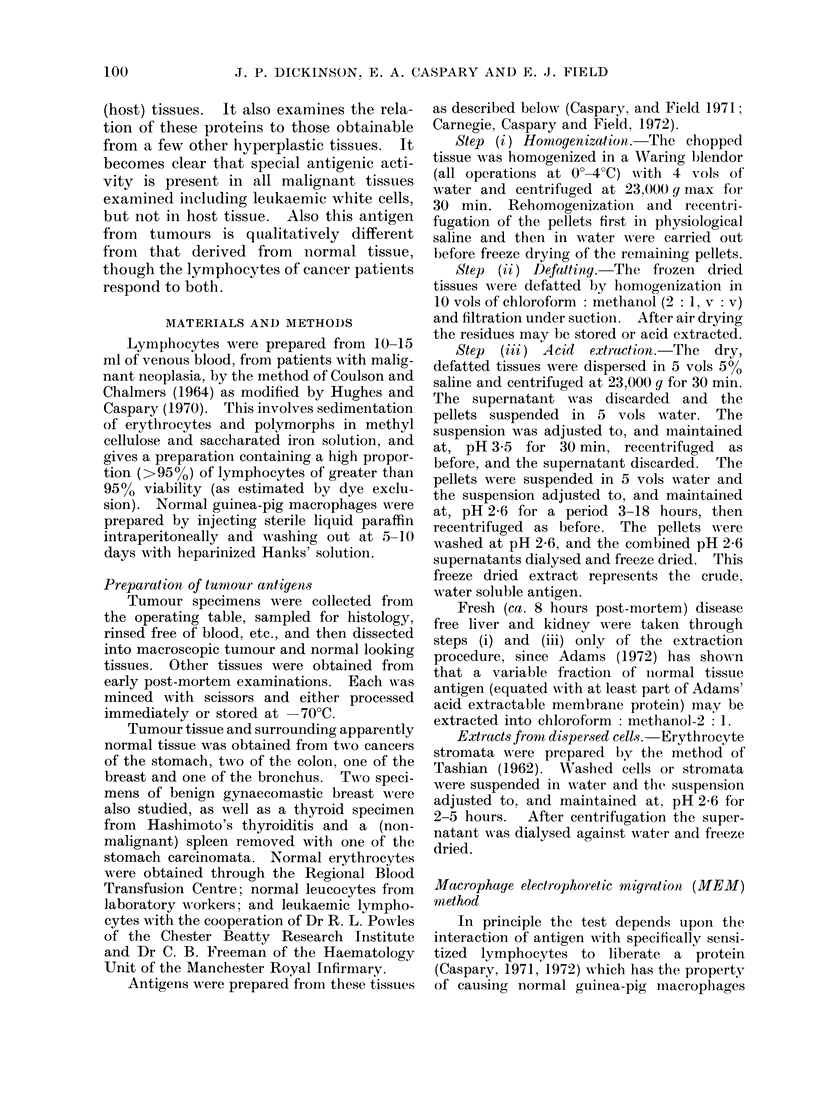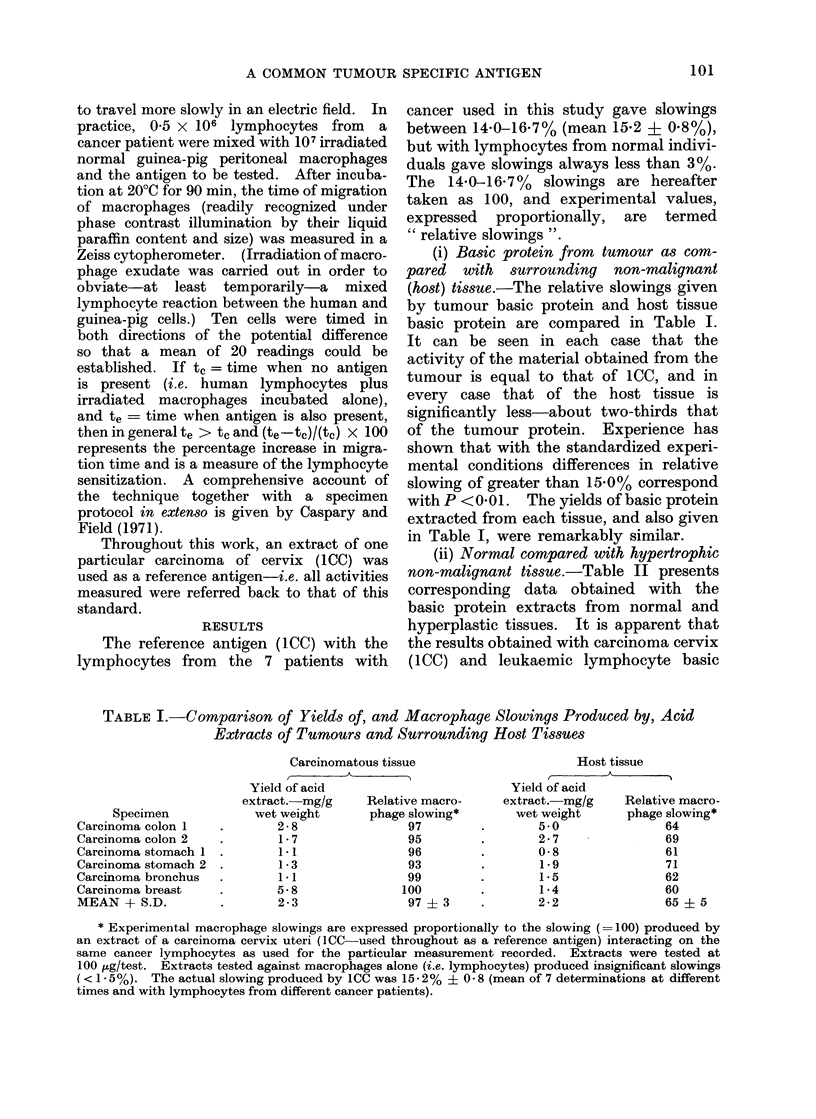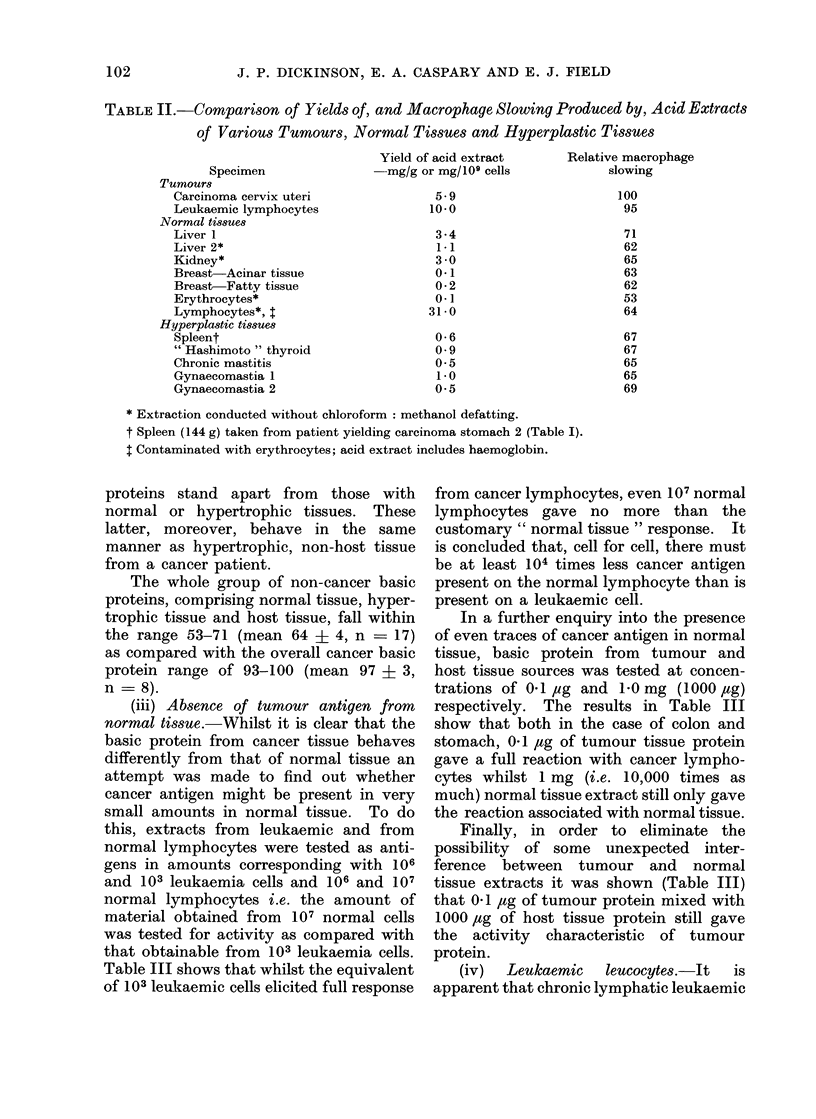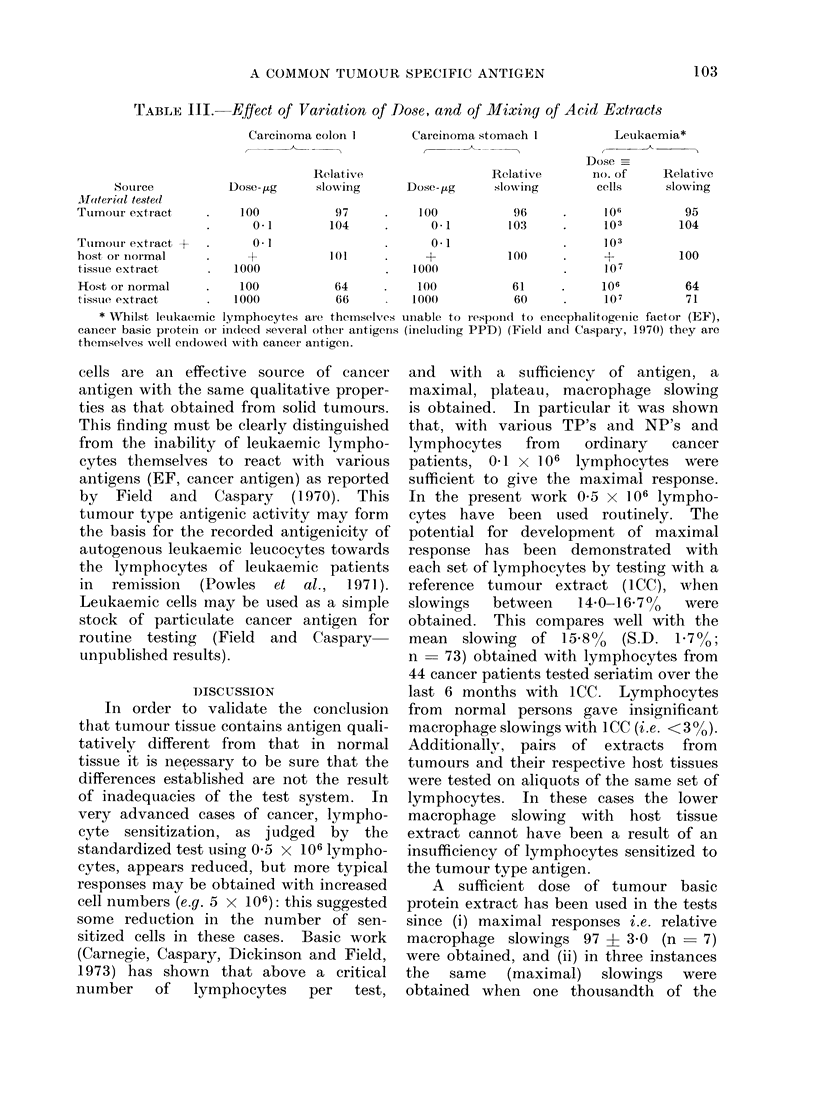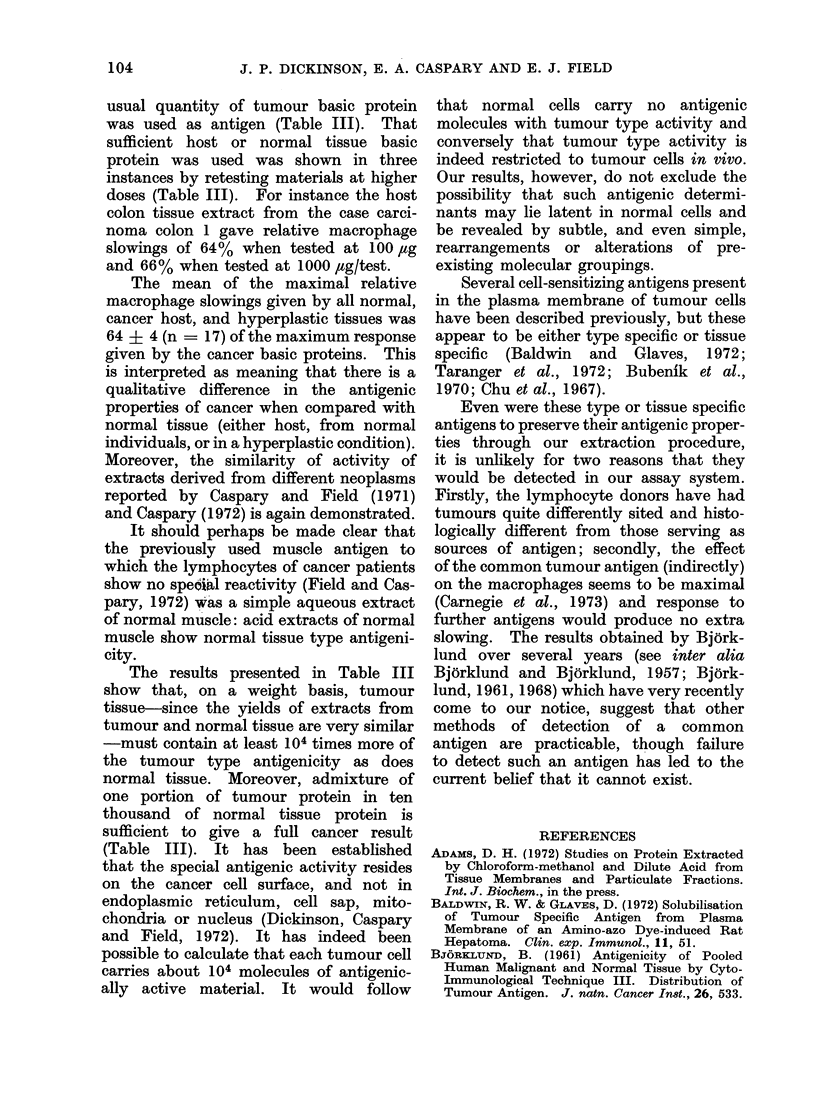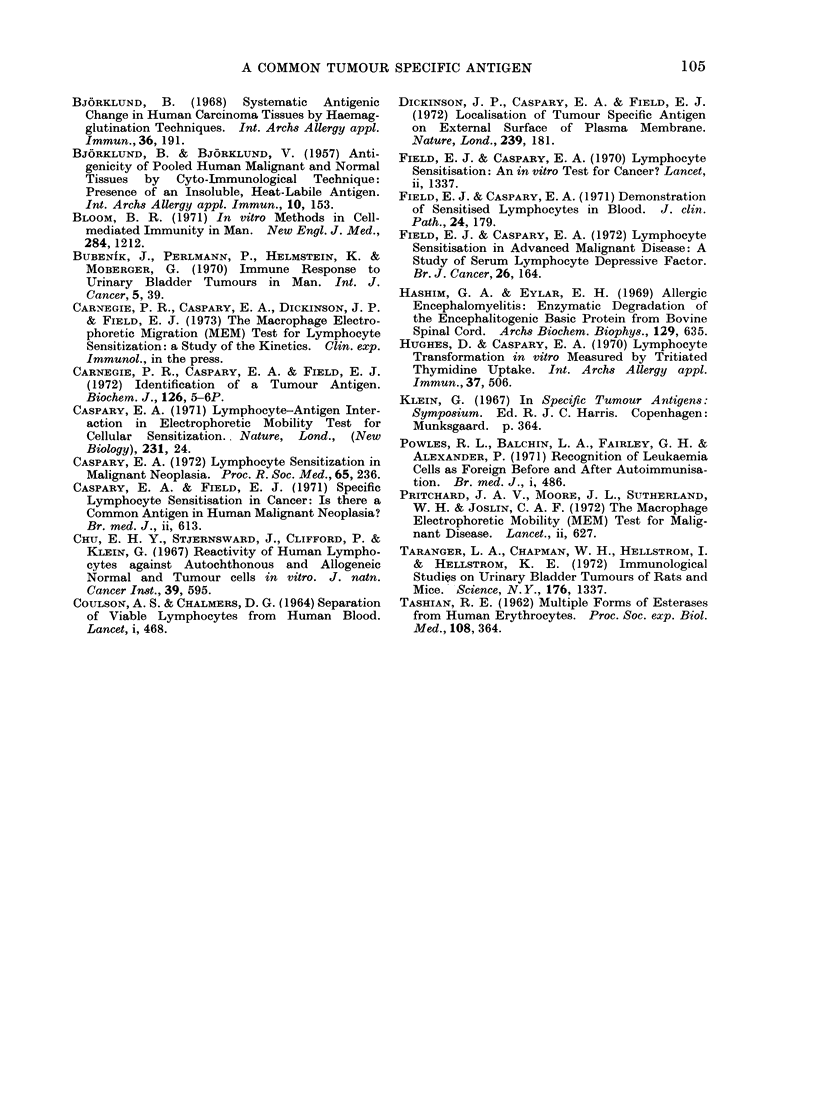# A Common Tumour Specific Antigen

**DOI:** 10.1038/bjc.1973.13

**Published:** 1973-02

**Authors:** J. P. Dickinson, E. A. Caspary, E. J. Field

## Abstract

(1) The antigenic activity of basic protein extracts of a variety of human tissues towards sensitized lymphocytes from cancer patients has been studied using the macrophage electrophoretic migration method.

(2) Basic protein prepared from human tumour tissue has antigenic properties which differ from basic protein prepared from normal tissues, from hyperplastic tissue and even tumour bearing host tissue.

(3) Tumour type antigenic molecules are at least 10^4^ times less numerous on normal cells than on tumour cells. If our reasoning is accepted then they are absent from normal cells, and thus restricted *in vivo* to malignant cells.

(4) Hyperplastic tissues (gynaecomastia, etc.) do not contain cancer basic protein.

(5) Human chronic lymphatic leukaemic leucocytes possess the same type of cancer antigenic activity as solid tumours. This antigenic activity is not shared by normal lymphocytes.


*Br. J. Cancer* (1973) **27**, 99

### A COMMON TUMOUR SPECIFIC ANTIGEN

### I. RESTRICTION *IN VIVO* TO MALIGNANT NEOPLASTIC TISSUE

J. P. DICKINSON, E. A. CASPARY AND E. J. FIELD

*From the Medical Research Council, Demyelinating Diseases Unit, Newcastle General Hospital, Newcastle upon Tyne NE4 6BE*

Received 12 September 1972. Accepted 20 October 1972

**Summary.—(1) The antigenic activity of basic protein extracts of a variety of human tissues towards sensitized lymphocytes from cancer patients has been studied using the macrophage electrophoretic migration method.**

**(2) Basic protein prepared from human tumour tissue has antigenic properties which differ from basic protein prepared from normal tissues, from hyperplastic tissue and even tumour bearing host tissue.**

**(3) Tumour type antigenic molecules are at least $10^4$ times less numerous on normal cells than on tumour cells. If our reasoning is accepted then they are absent from normal cells, and thus restricted *in vivo* to malignant cells.**

**(4) Hyperplastic tissues (gynaecomastia, etc.) do not contain cancer basic protein.**

**(5) Human chronic lymphatic leukaemic leucocytes possess the same type of cancer antigenic activity as solid tumours. This antigenic activity is not shared by normal lymphocytes.**

MANY tumour-produced antigens have been described, each of which appears to be characteristic only of a particular tumour or tumour type (see for example Baldwin and Glaves, 1972). We can find no published evidence, other than our own, that tumours may have a common but specific antigen, though quite recently it has been reported (Taranger *et al.*, 1972) that different tumour types from the same organ may share cell sensitizing antigens.

Hashim and Eylar (1969) from their detailed study of the immunogenic properties of the tryptophan peptide of the basic protein of myelin (encephalitogenic factor, EF) concluded that it provoked predominantly cellular sensitization and gave rise to little, if any, circulating antibody. Because of the general similarity between EF and the basic protein extractable from tumours (Caspary and Field, 1971), the latter might be expected to show a similar propensity to produce cell bound rather than humorally mediated reactions. Klein (1967) has emphasized the importance, in tumour immunology, of cell operated reactions as opposed to those dependent on free antibody. Study of cellular sensitization in man has been much hampered by the lack of a simple, reproducible and quantitative method of assessment (Bloom, 1971). The introduction of the macrophage electrophoretic migration (MEM) method (Field and Caspary, 1970) enabled some properties of the antigen(s) of human tumours to be established. Later (Caspary and Field, 1971) evidence was brought forward that there might be one antigen common to all human tumours. Whilst lymphocyte sensitization to EF in human malignant disease has now been independently confirmed (Pritchard *et al.*, 1972) the relationship between cancer basic proteins of different provenance remains to be clarified. The present work explores and compares the properties of basic protein extracted from a number of tumours with that derived from the surrounding normal

(host) tissues. It also examines the relation of these proteins to those obtainable from a few other hyperplastic tissues. It becomes clear that special antigenic activity is present in all malignant tissues examined including leukaemic white cells, but not in host tissue. Also this antigen from tumours is qualitatively different from that derived from normal tissue, though the lymphocytes of cancer patients respond to both.

### MATERIALS AND METHODS

Lymphocytes were prepared from 10–15 ml of venous blood, from patients with malignant neoplasia, by the method of Coulson and Chalmers (1964) as modified by Hughes and Caspary (1970). This involves sedimentation of erythrocytes and polymorphs in methyl cellulose and saccharated iron solution, and gives a preparation containing a high proportion ($>95\%$) of lymphocytes of greater than 95% viability (as estimated by dye exclusion). Normal guinea-pig macrophages were prepared by injecting sterile liquid paraffin intraperitoneally and washing out at 5–10 days with heparinized Hanks' solution.

### *Preparation of tumour antigens*

Tumour specimens were collected from the operating table, sampled for histology, rinsed free of blood, etc., and then dissected into macroscopic tumour and normal looking tissues. Other tissues were obtained from early post-mortem examinations. Each was minced with scissors and either processed immediately or stored at $-70°C$.

Tumour tissue and surrounding apparently normal tissue was obtained from two cancers of the stomach, two of the colon, one of the breast and one of the bronchus. Two specimens of benign gynaecomastic breast were also studied, as well as a thyroid specimen from Hashimoto's thyroiditis and a (non-malignant) spleen removed with one of the stomach carcinomata. Normal erythrocytes were obtained through the Regional Blood Transfusion Centre; normal leucocytes from laboratory workers; and leukaemic lymphocytes with the cooperation of Dr R. L. Powles of the Chester Beatty Research Institute and Dr C. B. Freeman of the Haematology Unit of the Manchester Royal Infirmary.

Antigens were prepared from these tissues as described below (Caspary, and Field 1971; Carnegie, Caspary and Field, 1972).

*Step (i) Homogenization.*—The chopped tissue was homogenized in a Waring blendor (all operations at 0°–4°C) with 4 vols of water and centrifuged at 23,000 *g* max for 30 min. Rehomogenization and recentrifugation of the pellets first in physiological saline and then in water were carried out before freeze drying of the remaining pellets.

*Step (ii) Defatting.*—The frozen dried tissues were defatted by homogenization in 10 vols of chloroform : methanol (2 : 1, v : v) and filtration under suction. After air drying the residues may be stored or acid extracted.

*Step (iii) Acid extraction.*—The dry, defatted tissues were dispersed in 5 vols 5% saline and centrifuged at 23,000 *g* for 30 min. The supernatant was discarded and the pellets suspended in 5 vols water. The suspension was adjusted to, and maintained at, pH 3·5 for 30 min, recentrifuged as before, and the supernatant discarded. The pellets were suspended in 5 vols water and the suspension adjusted to, and maintained at, pH 2·6 for a period 3–18 hours, then recentrifuged as before. The pellets were washed at pH 2·6, and the combined pH 2·6 supernatants dialysed and freeze dried. This freeze dried extract represents the crude, water soluble antigen.

Fresh (*ca.* 8 hours post-mortem) disease free liver and kidney were taken through steps (i) and (iii) only of the extraction procedure, since Adams (1972) has shown that a variable fraction of normal tissue antigen (equated with at least part of Adams' acid extractable membrane protein) may be extracted into chloroform : methanol-2 : 1.

*Extracts from dispersed cells.*—Erythrocyte stromata were prepared by the method of Tashian (1962). Washed cells or stromata were suspended in water and the suspension adjusted to, and maintained at, pH 2·6 for 2–5 hours. After centrifugation the supernatant was dialysed against water and freeze dried.

### *Macrophage electrophoretic migration (MEM) method*

In principle the test depends upon the interaction of antigen with specifically sensitized lymphocytes to liberate a protein (Caspary, 1971, 1972) which has the property of causing normal guinea-pig macrophages to travel more slowly in an electric field. In practice, $0 \cdot 5 \times 10^6$ lymphocytes from a cancer patient were mixed with $10^7$ irradiated normal guinea-pig peritoneal macrophages and the antigen to be tested. After incubation at 20°C for 90 min, the time of migration of macrophages (readily recognized under phase contrast illumination by their liquid paraffin content and size) was measured in a Zeiss cytopherometer. (Irradiation of macrophage exudate was carried out in order to obviate—at least temporarily—a mixed lymphocyte reaction between the human and guinea-pig cells.) Ten cells were timed in both directions of the potential difference so that a mean of 20 readings could be established. If $t_c$ = time when no antigen is present (*i.e.* human lymphocytes plus irradiated macrophages incubated alone), and $t_e$ = time when antigen is also present, then in general $t_e > t_c$ and $(t_e - t_c)/(t_c) \times 100$ represents the percentage increase in migration time and is a measure of the lymphocyte sensitization. A comprehensive account of the technique together with a specimen protocol *in extenso* is given by Caspary and Field (1971).

Throughout this work, an extract of one particular carcinoma of cervix (1CC) was used as a reference antigen—*i.e.* all activities measured were referred back to that of this standard.

### RESULTS

The reference antigen (1CC) with the lymphocytes from the 7 patients with cancer used in this study gave slowings between 14·0–16·7% (mean 15·2 ± 0·8%), but with lymphocytes from normal individuals gave slowings always less than 3%. The 14·0–16·7% slowings are hereafter taken as 100, and experimental values, expressed proportionally, are termed " relative slowings ".

(i) *Basic protein from tumour as compared with surrounding non-malignant (host) tissue.*—The relative slowings given by tumour basic protein and host tissue basic protein are compared in Table I. It can be seen in each case that the activity of the material obtained from the tumour is equal to that of 1CC, and in every case that of the host tissue is significantly less—about two-thirds that of the tumour protein. Experience has shown that with the standardized experimental conditions differences in relative slowing of greater than 15·0% correspond with $P < 0 \cdot 01$. The yields of basic protein extracted from each tissue, and also given in Table I, were remarkably similar.

(ii) *Normal compared with hypertrophic non-malignant tissue.*—Table II presents corresponding data obtained with the basic protein extracts from normal and hyperplastic tissues. It is apparent that the results obtained with carcinoma cervix (1CC) and leukaemic lymphocyte basic

TABLE I.—*Comparison of Yields of, and Macrophage Slowings Produced by, Acid Extracts of Tumours and Surrounding Host Tissues*

| | Carcinomatous tissue | | Host tissue | |
|---|---|---|---|---|
| Specimen | Yield of acid extract.—mg/g wet weight | Relative macrophage slowing* | Yield of acid extract.—mg/g wet weight | Relative macrophage slowing* |
| Carcinoma colon 1 | 2·8 | 97 | 5·0 | 64 |
| Carcinoma colon 2 | 1·7 | 95 | 2·7 | 69 |
| Carcinoma stomach 1 | 1·1 | 96 | 0·8 | 61 |
| Carcinoma stomach 2 | 1·3 | 93 | 1·9 | 71 |
| Carcinoma bronchus | 1·1 | 99 | 1·5 | 62 |
| Carcinoma breast | 5·8 | 100 | 1·4 | 60 |
| MEAN + S.D. | 2·3 | 97 ± 3 | 2·2 | 65 ± 5 |

* Experimental macrophage slowings are expressed proportionally to the slowing (=100) produced by an extract of a carcinoma cervix uteri (1CC—used throughout as a reference antigen) interacting on the same cancer lymphocytes as used for the particular measurement recorded. Extracts were tested at 100 μg/test. Extracts tested against macrophages alone (*i.e.* lymphocytes) produced insignificant slowings (< 1·5%). The actual slowing produced by 1CC was 15·2% ± 0·8 (mean of 7 determinations at different times and with lymphocytes from different cancer patients).

TABLE II.—*Comparison of Yields of, and Macrophage Slowing Produced by, Acid Extracts of Various Tumours, Normal Tissues and Hyperplastic Tissues*

| Specimen | Yield of acid extract —mg/g or mg/$10^9$ cells | Relative macrophage slowing |
|---|---|---|
| *Tumours* | | |
| Carcinoma cervix uteri | 5·9 | 100 |
| Leukaemic lymphocytes | 10·0 | 95 |
| *Normal tissues* | | |
| Liver 1 | 3·4 | 71 |
| Liver 2* | 1·1 | 62 |
| Kidney* | 3·0 | 65 |
| Breast—Acinar tissue | 0·1 | 63 |
| Breast—Fatty tissue | 0·2 | 62 |
| Erythrocytes* | 0·1 | 53 |
| Lymphocytes*, ‡ | 31·0 | 64 |
| *Hyperplastic tissues* | | |
| Spleen† | 0·6 | 67 |
| "Hashimoto" thyroid | 0·9 | 67 |
| Chronic mastitis | 0·5 | 65 |
| Gynaecomastia 1 | 1·0 | 65 |
| Gynaecomastia 2 | 0·5 | 69 |

* Extraction conducted without chloroform : methanol defatting.
† Spleen (144 g) taken from patient yielding carcinoma stomach 2 (Table I).
‡ Contaminated with erythrocytes; acid extract includes haemoglobin.

proteins stand apart from those with normal or hypertrophic tissues. These latter, moreover, behave in the same manner as hypertrophic, non-host tissue from a cancer patient.

The whole group of non-cancer basic proteins, comprising normal tissue, hypertrophic tissue and host tissue, fall within the range 53–71 (mean $64 \pm 4$, $n = 17$) as compared with the overall cancer basic protein range of 93–100 (mean $97 \pm 3$, $n = 8$).

(iii) *Absence of tumour antigen from normal tissue.*—Whilst it is clear that the basic protein from cancer tissue behaves differently from that of normal tissue an attempt was made to find out whether cancer antigen might be present in very small amounts in normal tissue. To do this, extracts from leukaemic and from normal lymphocytes were tested as antigens in amounts corresponding with $10^6$ and $10^3$ leukaemia cells and $10^6$ and $10^7$ normal lymphocytes *i.e.* the amount of material obtained from $10^7$ normal cells was tested for activity as compared with that obtainable from $10^3$ leukaemia cells. Table III shows that whilst the equivalent of $10^3$ leukaemic cells elicited full response from cancer lymphocytes, even $10^7$ normal lymphocytes gave no more than the customary "normal tissue" response. It is concluded that, cell for cell, there must be at least $10^4$ times less cancer antigen present on the normal lymphocyte than is present on a leukaemic cell.

In a further enquiry into the presence of even traces of cancer antigen in normal tissue, basic protein from tumour and host tissue sources was tested at concentrations of 0·1 $\mu$g and 1·0 mg (1000 $\mu$g) respectively. The results in Table III show that both in the case of colon and stomach, 0·1 $\mu$g of tumour tissue protein gave a full reaction with cancer lymphocytes whilst 1 mg (*i.e.* 10,000 times as much) normal tissue extract still only gave the reaction associated with normal tissue.

Finally, in order to eliminate the possibility of some unexpected interference between tumour and normal tissue extracts it was shown (Table III) that 0·1 $\mu$g of tumour protein mixed with 1000 $\mu$g of host tissue protein still gave the activity characteristic of tumour protein.

(iv) *Leukaemic leucocytes.*—It is apparent that chronic lymphatic leukaemic

TABLE III.—*Effect of Variation of Dose, and of Mixing of Acid Extracts*

| Source | Carcinoma colon 1 Dose-μg | Carcinoma colon 1 Relative slowing | Carcinoma stomach 1 Dose-μg | Carcinoma stomach 1 Relative slowing | Leukaemia* Dose ≡ no. of cells | Leukaemia* Relative slowing |
|---|---|---|---|---|---|---|
| *Material tested* | | | | | | |
| Tumour extract | 100 | 97 | 100 | 96 | $10^6$ | 95 |
| | 0·1 | 104 | 0·1 | 103 | $10^3$ | 104 |
| Tumour extract + host or normal tissue extract | 0·1 + 1000 | 101 | 0·1 + 1000 | 100 | $10^3$ + $10^7$ | 100 |
| Host or normal tissue extract | 100 | 64 | 100 | 61 | $10^6$ | 64 |
| | 1000 | 66 | 1000 | 60 | $10^7$ | 71 |

* Whilst leukaemic lymphocytes are themselves unable to respond to encephalitogenic factor (EF), cancer basic protein or indeed several other antigens (including PPD) (Field and Caspary, 1970) they are themselves well endowed with cancer antigen.

cells are an effective source of cancer antigen with the same qualitative properties as that obtained from solid tumours. This finding must be clearly distinguished from the inability of leukaemic lymphocytes themselves to react with various antigens (EF, cancer antigen) as reported by Field and Caspary (1970). This tumour type antigenic activity may form the basis for the recorded antigenicity of autogenous leukaemic leucocytes towards the lymphocytes of leukaemic patients in remission (Powles *et al.*, 1971). Leukaemic cells may be used as a simple stock of particulate cancer antigen for routine testing (Field and Caspary—unpublished results).

### DISCUSSION

In order to validate the conclusion that tumour tissue contains antigen qualitatively different from that in normal tissue it is necessary to be sure that the differences established are not the result of inadequacies of the test system. In very advanced cases of cancer, lymphocyte sensitization, as judged by the standardized test using $0·5 \times 10^6$ lymphocytes, appears reduced, but more typical responses may be obtained with increased cell numbers (*e.g.* $5 \times 10^6$): this suggested some reduction in the number of sensitized cells in these cases. Basic work (Carnegie, Caspary, Dickinson and Field, 1973) has shown that above a critical number of lymphocytes per test, and with a sufficiency of antigen, a maximal, plateau, macrophage slowing is obtained. In particular it was shown that, with various TP's and NP's and lymphocytes from ordinary cancer patients, $0·1 \times 10^6$ lymphocytes were sufficient to give the maximal response. In the present work $0·5 \times 10^6$ lymphocytes have been used routinely. The potential for development of maximal response has been demonstrated with each set of lymphocytes by testing with a reference tumour extract (1CC), when slowings between 14·0–16·7% were obtained. This compares well with the mean slowing of 15·8% (S.D. 1·7%; n = 73) obtained with lymphocytes from 44 cancer patients tested seriatim over the last 6 months with 1CC. Lymphocytes from normal persons gave insignificant macrophage slowings with 1CC (*i.e.* $<3\%$). Additionally, pairs of extracts from tumours and their respective host tissues were tested on aliquots of the same set of lymphocytes. In these cases the lower macrophage slowing with host tissue extract cannot have been a result of an insufficiency of lymphocytes sensitized to the tumour type antigen.

A sufficient dose of tumour basic protein extract has been used in the tests since (i) maximal responses *i.e.* relative macrophage slowings $97 \pm 3·0$ (n = 7) were obtained, and (ii) in three instances the same (maximal) slowings were obtained when one thousandth of the usual quantity of tumour basic protein was used as antigen (Table III). That sufficient host or normal tissue basic protein was used was shown in three instances by retesting materials at higher doses (Table III). For instance the host colon tissue extract from the case carcinoma colon 1 gave relative macrophage slowings of 64% when tested at 100 $\mu$g and 66% when tested at 1000 $\mu$g/test.

The mean of the maximal relative macrophage slowings given by all normal, cancer host, and hyperplastic tissues was $64 \pm 4$ ($n = 17$) of the maximum response given by the cancer basic proteins. This is interpreted as meaning that there is a qualitative difference in the antigenic properties of cancer when compared with normal tissue (either host, from normal individuals, or in a hyperplastic condition). Moreover, the similarity of activity of extracts derived from different neoplasms reported by Caspary and Field (1971) and Caspary (1972) is again demonstrated.

It should perhaps be made clear that the previously used muscle antigen to which the lymphocytes of cancer patients show no special reactivity (Field and Caspary, 1972) was a simple aqueous extract of normal muscle: acid extracts of normal muscle show normal tissue type antigenicity.

The results presented in Table III show that, on a weight basis, tumour tissue—since the yields of extracts from tumour and normal tissue are very similar —must contain at least $10^4$ times more of the tumour type antigenicity as does normal tissue. Moreover, admixture of one portion of tumour protein in ten thousand of normal tissue protein is sufficient to give a full cancer result (Table III). It has been established that the special antigenic activity resides on the cancer cell surface, and not in endoplasmic reticulum, cell sap, mitochondria or nucleus (Dickinson, Caspary and Field, 1972). It has indeed been possible to calculate that each tumour cell carries about $10^4$ molecules of antigenically active material. It would follow that normal cells carry no antigenic molecules with tumour type activity and conversely that tumour type activity is indeed restricted to tumour cells *in vivo*. Our results, however, do not exclude the possibility that such antigenic determinants may lie latent in normal cells and be revealed by subtle, and even simple, rearrangements or alterations of pre-existing molecular groupings.

Several cell-sensitizing antigens present in the plasma membrane of tumour cells have been described previously, but these appear to be either type specific or tissue specific (Baldwin and Glaves, 1972; Taranger *et al.*, 1972; Bubeník *et al.*, 1970; Chu *et al.*, 1967).

Even were these type or tissue specific antigens to preserve their antigenic properties through our extraction procedure, it is unlikely for two reasons that they would be detected in our assay system. Firstly, the lymphocyte donors have had tumours quite differently sited and histologically different from those serving as sources of antigen; secondly, the effect of the common tumour antigen (indirectly) on the macrophages seems to be maximal (Carnegie *et al.*, 1973) and response to further antigens would produce no extra slowing. The results obtained by Björklund over several years (see *inter alia* Björklund and Björklund, 1957; Björklund, 1961, 1968) which have very recently come to our notice, suggest that other methods of detection of a common antigen are practicable, though failure to detect such an antigen has led to the current belief that it cannot exist.

### REFERENCES

ADAMS, D. H. (1972) Studies on Protein Extracted by Chloroform-methanol and Dilute Acid from Tissue Membranes and Particulate Fractions. *Int. J. Biochem.*, in the press.

BALDWIN, R. W. & GLAVES, D. (1972) Solubilisation of Tumour Specific Antigen from Plasma Membrane of an Amino-azo Dye-induced Rat Hepatoma. *Clin. exp. Immunol.*, **11**, 51.

BJÖRKLUND, B. (1961) Antigenicity of Pooled Human Malignant and Normal Tissue by Cyto-Immunological Technique III. Distribution of Tumour Antigen. *J. natn. Cancer Inst.*, **26**, 533.

BJÖRKLUND, B. (1968) Systematic Antigenic Change in Human Carcinoma Tissues by Haemagglutination Techniques. *Int. Archs Allergy appl. Immun.*, **36**, 191.

BJÖRKLUND, B. & BJÖRKLUND, V. (1957) Antigenicity of Pooled Human Malignant and Normal Tissues by Cyto-Immunological Technique: Presence of an Insoluble, Heat-Labile Antigen. *Int. Archs Allergy appl. Immun.*, **10**, 153.

BLOOM, B. R. (1971) *In vitro* Methods in Cell-mediated Immunity in Man. *New Engl. J. Med.*, **284**, 1212.

BUBENÍK, J., PERLMANN, P., HELMSTEIN, K. & MOBERGER, G. (1970) Immune Response to Urinary Bladder Tumours in Man. *Int. J. Cancer*, **5**, 39.

CARNEGIE, P. R., CASPARY, E. A., DICKINSON, J. P. & FIELD, E. J. (1973) The Macrophage Electrophoretic Migration (MEM) Test for Lymphocyte Sensitization: a Study of the Kinetics. *Clin. exp. Immunol.*, in the press.

CARNEGIE, P. R., CASPARY, E. A. & FIELD, E. J. (1972) Identification of a Tumour Antigen. *Biochem. J.*, **126**, 5–6*P*.

CASPARY, E. A. (1971) Lymphocyte–Antigen Interaction in Electrophoretic Mobility Test for Cellular Sensitization. *Nature, Lond.*, (*New Biology*), **231**, 24.

CASPARY, E. A. (1972) Lymphocyte Sensitization in Malignant Neoplasia. *Proc. R. Soc. Med.*, **65**, 236.

CASPARY, E. A. & FIELD, E. J. (1971) Specific Lymphocyte Sensitisation in Cancer: Is there a Common Antigen in Human Malignant Neoplasia? *Br. med. J.*, ii, 613.

CHU, E. H. Y., STJERNSWARD, J., CLIFFORD, P. & KLEIN, G. (1967) Reactivity of Human Lymphocytes against Autochthonous and Allogeneic Normal and Tumour cells *in vitro*. *J. natn. Cancer Inst.*, **39**, 595.

COULSON, A. S. & CHALMERS, D. G. (1964) Separation of Viable Lymphocytes from Human Blood. *Lancet*, i, 468.

DICKINSON, J. P., CASPARY, E. A. & FIELD, E. J. (1972) Localisation of Tumour Specific Antigen on External Surface of Plasma Membrane. *Nature, Lond.*, **239**, 181.

FIELD, E. J. & CASPARY, E. A. (1970) Lymphocyte Sensitisation: An *in vitro* Test for Cancer? *Lancet*, ii, 1337.

FIELD, E. J. & CASPARY, E. A. (1971) Demonstration of Sensitised Lymphocytes in Blood. *J. clin. Path.*, **24**, 179.

FIELD, E. J. & CASPARY, E. A. (1972) Lymphocyte Sensitisation in Advanced Malignant Disease: A Study of Serum Lymphocyte Depressive Factor. *Br. J. Cancer*, **26**, 164.

HASHIM, G. A. & EYLAR, E. H. (1969) Allergic Encephalomyelitis: Enzymatic Degradation of the Encephalitogenic Basic Protein from Bovine Spinal Cord. *Archs Biochem. Biophys.*, **129**, 635.

HUGHES, D. & CASPARY, E. A. (1970) Lymphocyte Transformation *in vitro* Measured by Tritiated Thymidine Uptake. *Int. Archs Allergy appl. Immun.*, **37**, 506.

KLEIN, G. (1967) In *Specific Tumour Antigens: Symposium*. Ed. R. J. C. Harris. Copenhagen: Munksgaard. p. 364.

POWLES, R. L., BALCHIN, L. A., FAIRLEY, G. H. & ALEXANDER, P. (1971) Recognition of Leukaemia Cells as Foreign Before and After Autoimmunisation. *Br. med. J.*, i, 486.

PRITCHARD, J. A. V., MOORE, J. L., SUTHERLAND, W. H. & JOSLIN, C. A. F. (1972) The Macrophage Electrophoretic Mobility (MEM) Test for Malignant Disease. *Lancet.*, ii, 627.

TARANGER, L. A., CHAPMAN, W. H., HELLSTROM, I. & HELLSTROM, K. E. (1972) Immunological Studies on Urinary Bladder Tumours of Rats and Mice. *Science, N.Y.*, **176**, 1337.

TASHIAN, R. E. (1962) Multiple Forms of Esterases from Human Erythrocytes. *Proc. Soc. exp. Biol. Med.*, **108**, 364.